# Validation of HPLC and Enzyme-Linked Immunosorbent Assay (ELISA) Techniques for Detection and Quantification of Aflatoxins in Different Food Samples

**DOI:** 10.3390/foods9050661

**Published:** 2020-05-20

**Authors:** Sharaf S. Omar, Moawiya A. Haddad, Salvatore Parisi

**Affiliations:** Department of Nutrition and Food Processing, Faculty of Agricultural Technology, Al-Balqa Applied University, Al-Salt, P.O. Box Amman 36197, Jordan; haddad@bau.edu.jo (M.A.H.); drparisi@inwind.it (S.P.)

**Keywords:** aflatoxins, HPLC, ELISA, validation

## Abstract

Background: In Jordan as in other worldwide countries, mycotoxins are considered a serious national problem in food supplies. As a result, almost all nations are setting and adopting different regulations targeting the control of mycotoxins levels in the domestic food supply, including the problem of reliable sampling and analysis methods. Objective: It is necessary to improve and give evidence of analytical abilities of laboratories within Jordan and developing countries enabling them to monitor mycotoxins effectively in food to overcome non-tariff obstacles. Methods: We analyzed 40 samples from wheat, corn, dried fig and dried coffee beans for total aflatoxin content using High Pressure Liquid Chromatography (HPLC) and Enzyme Linked Immunesorbent Assay (ELISA) methods. Results: 40% of samples from wheat, 60% from corn, 30% from dried fig, and 50% from dried coffee beans were found positive when speaking of total aflatoxins, with average values between 1.14 and 4.12 μg/kg. Obtained results allow considering all tested food samples as fit for human consumption if compared with the labeled regulatory limit of allowed aflatoxins in the European Union. In detail, the limit of detection and the limit of quantification for methods used in this study were significantly lower than the maximum limits established by the European Union. Highlights: The procedure used in this study is suitable for detection of mycotoxins at very low concentration.

## 1. Introduction

Mycotoxins are considered secondary metabolites produced by toxigenic mold strains, and associated with severe health problems when ingested, inhaled or absorbed. These severe health complications include acute toxic, carcinogenic, mutagenic teratogenic and estrogenic effects. The five most important mycotoxins are ochratoxins, deoxynivalenol, zearalenone, fumonisins, and aflatoxins [1]. Infants and children are the most susceptible group to mycotoxins. However, many recent studies reported that the increasing number of infant milk and cereal-based foods exceeded the maximum acceptable level. Internationally, several studies and researches launched and insisted on the urgent need of surveying the contamination level of mycotoxins in baby milk and cereal-based formula, and also in a variety of foods and beverages worldwide [2,3,4,5,6,7].

Furthermore, since aflatoxins have proven toxic effects at very low concentrations there is a need for sensitive, reliable and accurate analytical methods for their determination. Different analytical methods for detection and quantification of aflatoxins are available in the literature. Many studies highlighted enzyme linked immunosorbent assay (ELISA) as the most frequently used technique for that detection of aflatoxins, followed by high performance liquid chromatography with fluorescence detector (HPLC-FLD) and liquid chromatography-tandem mass spectrometry (HPLC-MS/MS) [8,9,10]. Thin layer chromatography is less used technique comparing with other methods since it is reliable for detection of aflatoxins is low [11,12].

Many factors effect the growth of mold as well as the production of mycotoxins in food, such as climatic conditions, pest infestation, and poor harvest and storage practices. As the humidity increases during storage the growth of mold will increase and mycotoxins will be produces. One of the control methods for mycotoxins production is control of the storage conditions [13].

In Jordan as in other worldwide countries, mycotoxins are considered a serious national problem in the food supplies [14,15,16]. As a result, almost all nations are setting and adopting different regulations targeting the control of mycotoxins levels in the domestic food supply in addition to food exchanged internationally [17,18]. These strategies should force national and international authorities to call for further attention to develop standardized methods for required regulations and enforcing them into practice; that, of course, includes sampling and analysis methods. Therefore, it is vital to improve the analytical abilities of laboratories within Jordan and developing countries enabling them to monitor mycotoxins effectively in food to overcome non-tariff obstacles. In literature there is an unlimited availability of related data for a comparison of the validity of different methods—enzyme-linked immunosorbent assay (ELISA), high-performance liquid chromatography (HPLC), and gaschromatography/mass spectrometry (GC-MS) for mycotoxins detection [19,20].

## 2. Materials and Methods

### 2.1. Samples

We collected 40 different samples from local markets in Amman, Jordan, during 2017–2018 according to AOAC 977.16 [19], as shown in Table 1. Immediately after the collection, samples were transferred to the laboratory and prepared for subsequent HPLC and ELISA analyses according to AOAC 977.16 with relation to size reduction (by means of sanitized food cutters) and mixing (by means of normal disk mills).

### 2.2. Sample Analysis for HPLC

The method used for measuring total aflatoxins was followed according to Official Methods of Analysis (2000) 17th Ed., 1st Rev., AOAC INTERNATIONAL, Gaithersburg, MD, Method 991.31) with slight modifications [20]. We mixed 5 ± 0.05 g of ground sample with 30 mL of 80% methanol and shaked for 30 min at 120 rpm then filtered with paper (Whatman, St. Louis, MO, USA). We diluted 20 mL of filtered solution 2:1 with Phosphate Buffer Solution (PBS), then centrifuged it at 3500 rpm for 20 min, and subsequently filtered it (Whatman pore size 0.45Mm), We injected 50 ml from the extract into AflacleanTM immunoaffinity columns (LC Tech, Alpha, Germany) with flow rate of 0.5 mL per min. Subsequently, the fraction containing aflatoxins was slowly eluted with methanol and evaporated at 45 °C under nitrogen. Subsequently, 2 mL of the mobile phase of water:methanol:acetonitrile (60:30:15, *v*/*v*/*v*) were used to solve the residue, then filtered and injected into HPLC with fluorescent detection at wavelength 365 nm [21,22].

### 2.3. ELISA Determination

ELISA technique was used according to the manufacturing instruction (Romer Labs, Getzersdorf, Austria) with slight modifications [23]. About 5 g of a crushed sample was weighed and extracted with 10 mL of methanol–water mixture (7:3) to separate aflatoxin. The mixture was homogenized for 10 min at room temperature and then the resultant deposit was centrifuged. An aliqout (100 μL) of the supernatant was diluted with 600 μL of phosphate buffer then 50 μL an aliquot was added to the microwell, then incubated in dark place at room temperature for 60 min. The liquid was poured off the wells and tapped against absorbent paper to ensure liquid removing completely through the wells; then the conjugated enzyme (50 µL) was added into wells and incubated in the dark place at room temperature for 60 min. The washing process was repeated three times. After these processes, substrate (50 µL) and chromogen (50 µL) were added to each well, and incubated in the dark place at room temperature in 30 min. Stop reagent was added in each well and the absorbance at 450 nm was used [24,25].

### 2.4. Method Performance

The validity of quantification methods was assured according to the Commission Regulation (EC) No 401/2006 [26] by using certified reference material (CRM) and spiked samples. The validity of quantification methods was assured by using certified reference material (CRM) standards, which are needed for preparing a mixed stock solution in accordance with the EC 401/2006 document of the European Commission [14]. In detail, used CRM were: aflatoxins nos. A 6636, A 9887, A 0138, and A 0263 for AFB1, AFB2, AFG1, and AFG2, respectively (Sigma Aldrich, St. Louis, MO, USA). Adequate stock standard solutions of each of the four aflatoxins at 10 μg/mL were prepared in acetonitrile according to revised AOAC Method 971.22 [27].

First individual aflatoxin stock standard solution of 10 μg/mL was prepared by weighing 10 mg of each aflatoxin into a separate 100 mL-volumetric flask. Then 50 mL-acetonitrile was added, mixed and diluted to 100 ml with additional acetonitrile. Subsequently, 10 mL of the resulting solution was pipetted into another 100-mL volumetric flask and diluted to 100 mL with acetonitrile. The ultraviolet (UV) spectrum of each aflatoxin solution was recorded, and the concentration of aflatoxin solutions was determined by measuring absorbance (A) at wavelength of maximum absorption close to 360 nm and using equation in AOAC 971.22 (μg aflatoxin/mL = (A × MW × 1000)/ε) [28,29,30].

The second stock standard solution of 400 ng/mL AF (mixture of the 4 AFB1, B2, G1, and G2 at 200, 50, 100, and 50 ng/mL respectively) was prepared by adding an appropriate amount of each AF stock standard to the same volumetric flask and dilute to volume with acetonitrile. The 400 ng/mL AF second stock standard was used as the spiking solution for recovery study. All solutions were kept at −18 °C.

Working standard solutions of aflatoxins (AFS) were prepared daily in separated 10 mL volumetric flasks. The final aflatoxin concentrations of working standard solutions ranged from 0–8 ng/mL. Methanol (1:1) was used as a diluent. Standard curves were prepared for each of the AFS by using the working standard solutions containing the four AFs described. These solutions covered the ranges of 0.25–4 ng/mL for AFB1 (Aflatoxin B1), 0.0625–1 ng/mL for AFB2, 0.125–2 ng/mL for AFG1 (Aflatoxin G1) and 0.0625–1 ng/mL for AFG2 (Aflatoxin G2). The standard curves were obtained before the analysis according to working standard solutions. The plot for linearity was checked by examining the correlation coefficient (*R*^2^ > 0.99) of concentrations and responses. If the area response of the test solution was outside (higher) the standard range, the purified test extract was diluted with methanol–water (50 + 50, *v*/*v*) and re-injected into the LC column. The AFs were quantified by measuring the peak area at each AF retention time and comparing it with the relevant standard curve. Peak area (response, Y-axis) of each AF standard versus concentration (ng/mL, X-axis) was plotted and the slope (S) and Y-intercept (a) was determined. The level of toxins in test samples was calculated by using the equation shown below, where R is the peak area obtained for the test solution, and V is the final volume (mL) of the injected test solution. F is the dilution factor. F is 1 when V is 3 mL. W is 1 g test sample passed through the immunoaffinity column. Total AF is the sum of AFB1, AFB2, AFG1, and AFG2.

Toxin, µg/kg = ([(R − a)/S] × V/W) × F

All solutions and reagents used were of analytical (HPLC) and certified grades. Each procedure was repeated at least three times and results were averaged to enhance precision and accuracy. Appropriate amount of mycotoxin standards were spiked to mycotoxins-free food samples with replicates at three concentration levels (0, 0.5, and 1.0 μg/kg). The statistical measurements for the applied ELISA, and other chromatographic levels were calculated and expressed according to Technical Report CEN/TR 16059:2010 from European Committee for Standardization [31]. Limits of detection (LOD) and quantification (LOQ) were calculated by adding standard solution of mycotoxins into samples with decreasing concentrations, and then subject to extraction and quantification to measure the lowest detectable concentration (LOD) and the lowest quantifiable concentration (LOQ). Relative Standard Deviation (RSD) was calculated to express the precision according to the repeatability of the recovery experiments for each concentration (0, 0.5, and 1.0 μg/kg) [31,32,33].

### 2.5. Statistical Analysis

Duncan’s multiple comparison tests were used for Analysis of Variance (ANOVA) by means of STATISTICA software version 12.35 (Hamburg, Germany). Obtained results (*p* < 0.05) were regarded as significant.

## 3. Results and Discussions

Table 2, Table 3 and Table 4 show LOD and LOQ values for methods used in this study. Values were significantly lower than the maximum limits established by European Union for wheat (4.0 μg/kg), coffee beans (5.0 μg/kg), corn (5.0 μg/kg), and dried figs (4.0 μg/kg) [22]. Accordingly, all methods used in this study were suitable for detection of mycotoxins at very low concentration [28]. Furthermore, the mean recovery (recovery percentage) was determined at three concentration levels (0.05, 0.5, and 1.0 μg/kg). Recovery values (Table 2) confirmed that the optimal recovery was obtained in both HPLC and ELISA evaluations. In addition, ELISA method showed the higher recovery values if compared with recovery values obtained by HPLC (Table 3 and Table 4). The calculated values of recovery percentage for all methods used were in the range 74.1–96.9% (Table 2, Table 3 and Table 4). This result will give suitable validity for all methods used in this study. In addition, obtained results confirm previous studies. In summary, ELISA tests gave recovery values closer to 100% in comparison to chromatographic methods [10]. Precision—expressed under repeatability conditions—gave RSD values within the range of 3.5–12.4 and 3.5–13.1 for ELISA and HPLC, respectively. These values coincide with the mentioned criteria of RSD ≤15% which indicated a good precision of the methods.

**Figure 1:**
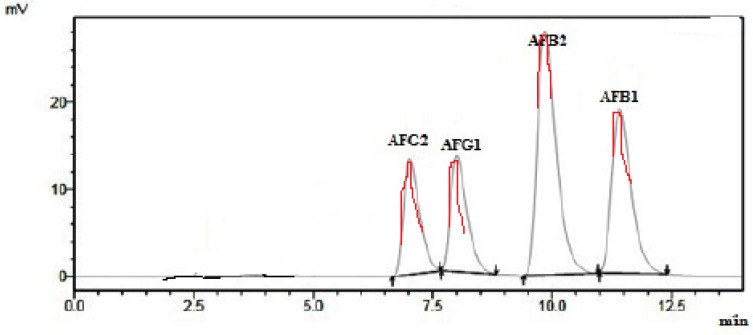
Chromatogram of spiked corn sample with AFB1, AFB2, AFG1 and AFG2.

In present study, Figure 1 shows a good chromatography for AFB1, AFB2, AFG1 and AFG2 with acceptable baseline and resolution for each aflatoxin. The aflatoxin peaks of blank and spiked samples were well separated and there were foreign peaks interfered with aflatoxin peak. This method exhibited good specificity and selectivity [31].

Table 5 shows total aflatoxin contents in forty samples from different food items (wheat, corn, dried fig, and dried coffee beans) using HPLC and ELISA. It was found that 40% of samples from wheat, 60% from corn, 30% from dried figs, and 50% from dried coffee beans were positive samples. The obtained mean values of total aflatoxins were 2.57, 2.19, 4.12, 3.87, 2.21, 2.13, 1.14, and 1.47 for wheat, corn, dried fig and dried coffee beans, respectively. With relation to European Union limits concerning the presence of aflatoxins in food items (4, 5, 4, and 5 µg/kg for wheat, corn, dried fig and dried coffee beans, respectively), all tested food samples were judged fit for human consumption. It can be also observed (Table 3) that ELISA method did not show fake positive results: detection of mycotoxins using ELISA method were confirmed by HPLC method. Furthermore, the values of mycotoxins using ELISA gave slightly higher values in comparison to values obtained by HPLC [34].

It was also found that the correlation coefficient between ELISA and others chromatographic methods depended on specificity and reproducibility of the used monoclonal antibody. In detail, it was reported that food matrix has strong effect on correlation between used methods. It was found that there was a good correlation between chromatographic methods and ELISA for mycotoxin determination in peanut and oilseeds, with low correlation for cereals and grains [29,34]. The present study shows the validity of all methods for determination of mycotoxins in foods; the choice of the method depends on the availability of equipment and the type of food samples. In this study, ELISA method was less-time consuming and less expensive because there was no need for complicated sample preparation procedures [35].

## 4. Conclusions

In summary, this research demonstrates the validation of ELISA and HPLC for detection and quantification of mycotoxins in different food samples available in the Jordanian market. It was found that both ELISA and HPLC are suitable for mycotoxins analysis. There was a strong correlation between two used methods. The selection of analytical methods depends basically on the availability and number of samples. ELISA method has some advantages such as less time needed and simplicity. On the other hand, HPLC—as with other chromatographic methods—is more accurate and specific than ELISA.

## Figures and Tables

**Table 1:** Forty different samples have been collected from local markets in Amman, Jordan, during 2017-2018. The analytical plan included enzyme-linked immunosorbent assay (ELISA) and high-performance liquid chromatography (HPLC) analyses on all samples.

Item	Number of Samples	Analytical Parameter	Method Used
Wheat	10	Total aflatoxins	ELISA, HPLC
Corn	10	Total aflatoxins	ELISA, HPLC
Dried fig	10	Total aflatoxins	ELISA, HPLC
Dried coffee beans	10	Total aflatoxins	ELISA, HPLC

**Table 2:** Statistical parameters for the determination of total aflatoxins in food samples by using HPLC and ELISA techniques.

Food Sample	Technique	LOD µg/kg	LOQ µg/kg	CRM	Recovery (%)			RSD		
					0.05 µ/kg	0.5 µ/kg	1.0 µ/kg	0.05 µ/kg	0.5 µ/kg	1.0 µ/kg
Wheat	ELISA	0.05	0.09	105.2	92.3	94.1	96.9	08.2	06.1	05.3
	HPLC	0.01	0.04	88.1	84.1	84.3	87.5	11.1	10.3	09.3
Corn	ELISA	0.10	0.40	104.1	95.2	98.9	93.1	9.3	6.4	7.3
	HPLC	0.05	0.09	87.5	80.1	82.6	80.8	10.1	9.1	11.1
Dried fig	ELISA	0.02	0.40	111.3	88.2	80.9	84.9	8.2	5.1	4.9
	HPLC	0.03	0.45	85.4	84.1	79.6	82.3	12.1	10.4	8.4
Coffee beans	ELISA	0.071	0.12	103.2	83.2	88.1	83.5	9.1	6.1	5.1
	HPLC	0.03	0.32	79.1	74.1	80.6	82.2	13.1	11.3	10.4

LOD: Limit of Detection, LOQ; Limit of Quantification, CRM: Certified Refernce Material, RSD: Relative standard deviation.

**Table 3:** Statistical parameters for the determination of AFB1, AFB2, AFG1, and AFG2 in different food samples by using ELISA techniques.

**Toxins**	**Wheat**							
	LOD µg/kg	LOQ µg/kg	Recovery (%)			RSD		
			0.05 µ/kg	0.5 µ/kg	1.0 µ/kg	0.05 µ/kg	0.5 µ/kg	1.0 µ/kg
AFB1	0.05	0.12	90.1	91.6	94.3	7.6	7.7	4.3
AFB2	0.04	0.09	88.5	89.5	91.4	6.4	5.4	3.6
AFG1	0.06	0.15	92.4	94.6	96.1	5.3	6.6	7.9
AFG2	0.07	0.13	89.5	90.5	92.1	5.6	5.4	8.5
	**Corn**							
	LOD µg/kg	LOQ µg/kg	Recovery (%)			RSD		
			0.05 µ/kg	0.5 µ/kg	1.0 µ/kg	0.05 µ/kg	0.5 µ/kg	1.0 µ/kg
AFB1	0.21	0.51	76.2	77.2	87.5	6.5	7.5	3.5
AFB2	0.09	0.12	84.3	87.9	88.7	4.4	3.5	6.6
AFG1	0.58	0.91	93.1	88.9	91.4	5.6	8.5	7.8
AFG2	0.65	0.98	83.8	88.5	90.5	7.8	4.9	7.7
	**Dried fig**							
	LOD µg/kg	LOQ µg/kg	Recovery (%)			RSD		
			0.05 µ/kg	0.5 µ/kg	1.0 µ/kg	0.05 µ/kg	0.5 µ/kg	1.0 µ/kg
AFB1	0.06	0.41	79.4	81.2	85.4	5.7	7.9	6.6
AFB2	0.12	0.31	83.4	88.5	81.4	7.7	6.9	5.6
AFG1	0.23	0.32	85.3	89.4	84.3	6.8	4.6	4.8
AFG2	0.16	0.21	87.6	90.5	85.0	5.5	3.5	3.9
	**Coffee beans**							
	LOD µg/kg	LOQ µg/kg	Recovery (%)			RSD		
			0.05 µ/kg	0.5 µ/kg	1.0 µ/kg	0.05 µ/kg	0.5 µ/kg	1.0 µ/kg
AFB1	0.06	0.12	85.4	90.1	92.1	6.8	4.6	3.5
AFB2	0.07	0.13	87.5	91.1	94.3	4.9	5.9	6.5
AFG1	0.06	0.09	88.5	89.9	89.3	12.4	3.4	4.4
AFG2	0.12	0.15	89.5	93.1	95.5	5.6	9.6	8.5

AFB1: Aflatoxin B1, AFB2: Aflatoxin B2, AFG1: aflatoxin G1, AFG2: Aflatoxin G2.

**Table 4:** Statistical parameters for the determination of AFB1, AFB2, AFG1, and AFG2 in different food samples by using HPLC techniques.

Toxins	**Wheat**							
	LOD µg/kg	LOQ µg/kg	Recovery (%)			RSD		
			0.05 µ/kg	0.5 µ/kg	1.0 µ/kg	0.05 µ/kg	0.5 µ/kg	1.0 µ/kg
AFB1	0.04	0.09	90.1	88.6	90.3	8.6	8.6	5.5
AFB2	0.02	0.05	88.5	82.5	88.3	7.7	7.6	4.4
AFG1	0.04	0.11	92.4	90.6	90.5	6.5	8.9	8.9
AFG2	0.06	0.09	89.5	83.5	89.5	9.8	9.8	9.5
	**Corn**							
	LOD µg/kg	LOQ µg/kg	Recovery (%)			RSD		
			0.05 µ/kg	0.5 µ/kg	1.0 µ/kg	0.05 µ/kg	0.5 µ/kg	1.0 µ/kg
AFB1	0.19	0.41	66.7	72.3	84.4	7.7	6.7	8.7
AFB2	0.05	0.09	80.5	83.2	81.3	6.9	7.8	9.9
AFG1	0.32	0.82	90.1	83.9	90.4	9.4	9.6	8.0
AFG2	0.43	0.65	80.1	81.4	89.3	8.8	5.0	4.6
	**Dried fig**							
	LOD µg/kg	LOQ µg/kg	Recovery (%)			RSD		
			0.05 µ/kg	0.5 µ/kg	1.0 µ/kg	0.05 µ/kg	0.5 µ/kg	1.0 µ/kg
AFB1	0.05	0.31	77.2	77.5	80.5	3.5	12.1	9.9
AFB2	0.11	0.20	80.5	81.4	77.9	8.8	9.9	7.6
AFG1	0.16	0.23	79.0	80.9	79.5	9.9	7.9	8.0
AFG2	0.08	0.12	81.3	87.5	80.5	7.0	5.5	4.9
	**Coffee beans**							
	LOD µg/kg	LOQ µg/kg	Recovery (%)			RSD		
			0.05 µ/kg	0.5 µ/kg	1.0 µ/kg	0.05 µ/kg	0.5 µ/kg	1.0 µ/kg
AFB1	0.04	0.09	82.6	88.4	90.1	7.0	9.0	5.5
AFB2	0.06	0.11	81.1	84.3	89.2	6.6	4.9	7.7
AFG1	0.09	0.07	83.2	81.9	88.4	10.6	4.0	8.9
AFG2	0.09	0.13	81.9	90.4	88.5	6.8	10.3	9.0

**Table 5:** Total aflatoxin levels in different food samples by using ELISA and HPLC. No differences have been observed between the ELISA and HPLC results (*p* > 0.05). SD is for: standard deviation.

Food	Method	Number	Positive Samples	Mean ± SD µ/kg	Total Aflatoxin Limits According to EC/Codex Regulation (17)
Wheat	ELISA	10	40%	2.57 ± 1.15 ^a^	4 µg/kg
	HPLC	10		2.19 ± 1.65 ^a^	
Corn	ELISA	10	60%	4.12 ± 1.05 ^a^	5 µg/kg
	HPLC	10		3.87 ± 1.17 ^a^	
Dried fig	ELISA	10	30%	2.21 ± 2.25 ^a^	4 µg/kg
	HPLC	10		2.13 ± 2.07 ^a^	
Dried coffee beans	ELISA	10	50%	1.14 ± 1.09 ^a^	5 µg/kg
	HPLC	10		1.47 ± 1.88 ^a^	

Values are mean ± SD of three replicates; means with different letters within a column are significantly different Least Significant Difference (LSD) < 0.05.
